# Comparison of Variants of Uncertain Significance in Three Regions of the Human Glucokinase Protein Using In Vitro and In Silico Analyses

**DOI:** 10.7759/cureus.68638

**Published:** 2024-09-04

**Authors:** Carter Gay, Shelby Watford, Eric B Johnson

**Affiliations:** 1 Medical School, Alabama College of Osteopathic Medicine, Dothan, USA; 2 Anatomy and Molecular Medicine, Alabama College of Osteopathic Medicine, Dothan, USA

**Keywords:** maturity-onset diabetes of the young 2, mody2, molecular dynamics simulation, variant of uncertain significance, glucokinase

## Abstract

There is a growing field of research focusing on the bioinformatic analysis of human genetic variation and the associated diseases. To study how well in vitro testing of purified proteins compares to bioinformatic variant prediction, we chose to analyze glucokinase (GCK) missense variations between residues 119-132, 257-262, and 412-427. These regions contained a large number of variants of uncertain significance (VUS) as well as a few pathogenic variants to use for comparison. We compared experimentally produced Vmax values from purified GCK variant proteins to predictive methods such as molecular dynamics simulation, ConSurf, iStable, the evolutionary model of variant effect (EVE), PredictSNP, and calculated binding energy. After determining which variants are pathogenic or benign based on experimental results or previous genetic studies, we found that ConSurf was the best at predicting pathogenicity. Interestingly, one VUS, D262N, showed an increase in activity and thus was difficult to interpret as pathogenic or benign. This study is an attempt to provide a framework for the utility of missense variant predictive programs.

## Introduction

Autosomal dominant pathogenic human genetic variants of glucokinase (Hexokinase 4, GCK) cause maturity-onset diabetes of the young 2 (MODY 2) [1]. MODY 2 is characterized by "mild, asymptomatic hyperglycemia in nonobese children, adolescents, and young adults who have a prominent family history of diabetes" [1]. These patients often develop type II diabetes mellitus later in life [1].

GCK is a member of the hexokinase family of genes and thus catalyzes the phosphorylation of glucose in the first step of glycolysis. GCK is different from other hexokinases in that it is not inhibited by high glucose-6-phosphate concentrations. Thus, it is used by the pancreatic islet beta cells and the liver to monitor blood glucose levels, allowing the coupling of insulin release proportionally to circulating glucose levels [2,3]. Pathogenic variants of GCK cause hyperglycemia because they have a higher threshold of glucose concentration needed for insulin secretion. Residues Glu256, Glu290, Thr168, Lys169, Asn204, and Asp205 are involved in glucose binding [4]. Alpha helix 13 in GCK (residues 442-464) also appears to play a role in the transition between open and closed states of the enzyme [4].

GCK variations have also been shown to be associated with type II diabetes mellitus. In fact, GCK agonists are currently being investigated as potential treatments for this condition due to their ability to improve sensitivity to glucose and enhance insulin release [5-9].

In this report, we use the YASARA structure program to run molecular dynamics simulations and perform docking experiments [10]. The molecular dynamics simulations replicate the movements of the variant proteins in solution over a period of time on the order of nanoseconds [11]. Changes in the position of residues during the simulation can be detected by changes in root-mean-square deviation (RMSD). Global changes in protein structure can be detected with total RMSD, while the change in position of specific residues can be detected with residue RMSD. The YASARA structure program also calculates the binding energy of the ligand or substrate using a docking macro [12].

There are many web-based predictive programs that can be used to determine variant pathogenicity [13]. In this report, we use ConSurf, iStable, the evolutionary model of variant effect (EVE), and PredictSNP. ConSurf measures conservation at each residue by comparing residue variation in homologous proteins. The program then provides a substitution score to represent how easily that residue can be substituted, as well as a list of residues that are permissible substitutes at that position [14]. iStable predicts the stability of a protein by using a collection of predictive programs and creates a consensus score for stability [15]. This program has been used before for variant analysis [16]. It was developed to identify more stable protein variants for use in industrial applications [15]. EVE is a predictive program that uses deep generative models using evolutionary sequence constraints [17]. PredictSNP is a consensus classifier that combines the outputs of several predictive programs for the determination of the pathogenicity of a variation with an associated confidence score [18].

The main objective of this research is to compare in vitro data to a select group of predictive programs to gain insight into accurate prediction of variants of uncertain significance (VUS) pathogenicity with the long-term goal of being able to predict disease risk in patients. We chose GCK as a model protein that is easily assayable in vitro compared to the in silico analysis. The secondary objective is to analyze a set of known VUS in GCK to contribute to the knowledge of these specific variants in patients.

## Materials and methods

GCK variant plasmid construction

The FASTA file P35557 obtained from UniProt [19] was used as the template for the cDNA [20]. Previous publications show that GCK tolerates an amino-terminal tag [21,22]. The full-length sequences of the native and variant proteins were submitted to Twist Biosciences Inc. (San Francisco, California) to be cloned into the pET-28a(+) vector using the Sac1-HindIII site. This vector codes for six amino-terminal histidines for nickel-column purification. The primary protein sequences submitted included a stop codon to prevent the addition of a carboxyl-terminal tag. The nucleotide sequence was optimized for *Escherichia coli* (*E. coli*) expression.

GCK protein expression and purification

HMS174 (DE3) competent cells (Millipore Sigma, St. Louis, Missouri) were transformed with the expression vectors using standard techniques. Bacterial clones were grown in terrific broth (Thermo Fisher, Waltham, Massachusetts) to an OD600 of 0.6. Isopropyl-β-D-1-thiogalactopyranoside (IPTG) (Millipore Sigma, St. Louis, Missouri) was added to the cultures for a final concentration of 1 mM, and the bacteria were cultured with shaking at 225 RPM overnight at room temperature. The bacteria were centrifuged at 4,000 g for 20 minutes at 4 °C. The protein was purified using the manufacturer's recommended protocol for native protein purification with the Ni-NTA Spin Kit (Qiagen, Tegelen, Netherlands). Protein purity and concentration were determined by running varying dilutions of purified protein and bovine serum albumin standards on an SDS-PAGE gel and staining with Coomassie reagent using standard protocols. Band intensity was measured at 800 nm using ImageStudio for the Li-Cor Odyssey CLx Imaging System (Li-Cor, Lincoln, Nebraska).

GCK protein activity assay

The GCK activity assay was established previously [21]. The isolated proteins were diluted to a common concentration using elution buffer and aliquoted for 1 µg per reaction. The reaction mix was added to the protein to make the following final concentrations: 100 mM Tris-HCl pH 7.4 (Boston Bioproducts, Milford, Massachusetts), 5 mM MgCl (Millipore Sigma, St. Louis, Missouri), 14 mM β-mercaptoethanol (Thermo Fisher, Waltham, Massachusetts), 0.1% bovine serum albumin (Millipore Sigma, St. Louis, Missouri), 150 mM KCl (Thermo Fisher, Waltham, Massachusetts), 0.4 mM NADP (Millipore Sigma, St. Louis, Missouri), 0.2 units/mL glucose-6-phosphate dehydrogenase (Millipore Sigma, St. Louis, Missouri), 100 mM glucose (Alfa Aesar, Haverhill, Massachusetts), and 5 mM ATP (Alfa Aesar, Haverhill, Massachusetts). The solutions were mixed with pipetting and transferred to a UV-transparent microplate (Corning, Corning, New York), and the absorbance at 340 nm was read using an Agilent Bio Tek plate reader. The absorbance was measured every three minutes for a 30-minute range, and the Vmax was calculated using the Gen5 (Agilent Bio Tek, Santa Clara, California) program. Three assays in triplicate were run for each purified variant.

Molecular modeling and molecular dynamics simulation

All GCK homology modeling and molecular dynamics simulations were performed using the YASARA structure program [10,23,24]. The GCK primary sequence FASTA file NP_000153.1 was obtained from NCBI and used as the input for the homology modeling function in the YASARA structure. The resulting structure contained all 465 residues and a glucose molecule in the active site. The variant residues were changed in the structure to produce the variant Protein Data Bank (PDB) files. Each file was corrected using the em_runclean macro. Replicates of each file were produced by running an energy minimization and saving the resulting file.

For the molecular dynamics simulation, the hydrogen bonding and protonation were optimized to a pH of 7.4 [25]. NaCl ions were added to a concentration of 0.9%. The simulation was run for 20 ns using the AMBER14 force field [26] for the solute and TIP3P for water. The intramolecular timestep was 1.3333 fs, and the intermolecular timestep was 4.0 fs. A temperature of 298 K and pressure of 1 atm was used. After inspection of the solute RMSD as a function of simulation time, the first 10 ns were considered equilibration time and excluded from further analysis. The data were obtained by running the md_analyze and md_analyzeres macros [10].

Predictive programs

The following describes how data were obtained from each of the variant predictive programs. The PDB identifier 3F9M was inputted into the ConSurf program [14] and iStable 2.0 program [15]. The UniProt gene code "GCK (HXK4_HUMAN, P35557)" was entered into the EVE website [20]. The GCK primary sequence FASTA file NP_000153.1 was obtained from NCBI and used as the template for input into PredictSNP [18].

Glucose docking

Each of the variant replicate PDB files used in the molecular dynamics simulations was used for the docking experiments using the protocol suggested in the YASARA structure manual. Each PDB file was converted to a .sce file in which the ligand, glucose, has been made a separate object from the protein and surrounded by a simulation cell. The docking experiment was performed by running the dock_runlocal macro [27].

## Results

Selection of GCK variants to analyze

**Figure 1:**
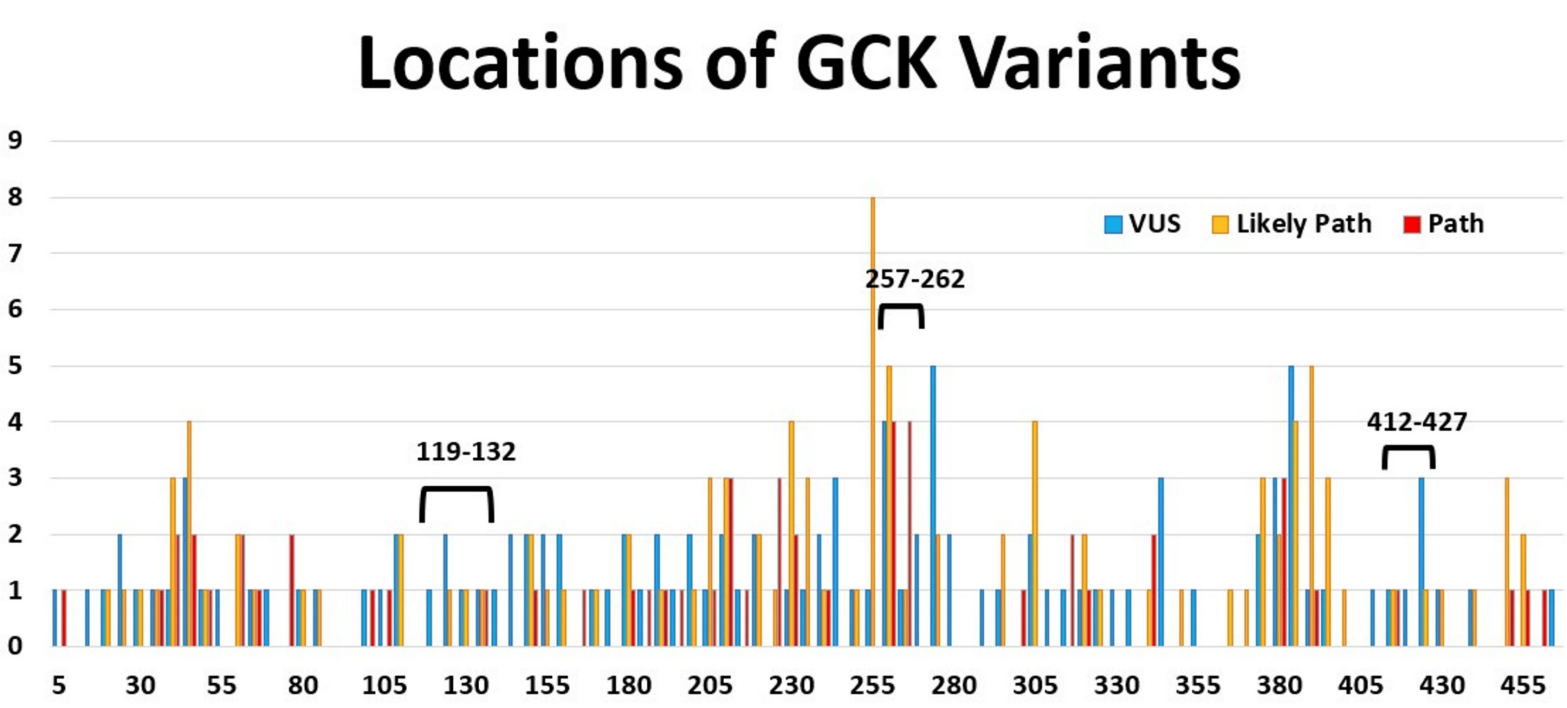
Frequency of glucokinase variants of uncertain significance on primary structure. A graph of the distribution of variants of uncertain significance (blue), pathogenic variants (red), and likely pathogenic (orange) variants, showing the relative location of each variant. The positions are grouped in bins of five residues. The bracketed regions indicate where the tested variants of uncertain significance were located. The 119-132 and the 412-427 regions have a low number of pathogenic/likely pathogenic variants, while the 257-262 region has a high number of pathogenic/likely pathogenic variants. GCK: glucokinase; VUS: variants of uncertain significance; Path: pathogenic.

We initially wanted to test if VUS in parts of a protein with few pathogenic variants have a higher likelihood of being benign. This is based on the idea that important parts of a protein will have a higher frequency of pathogenic variants, and thus, VUS in that area will also likely be pathogenic. To test this hypothesis, we selected two experimental regions and one control region of the GCK protein. The experimental regions had a relatively low frequency of pathogenic variants, while the control region had a high frequency of pathogenic variants, including some variants that have been repeatedly demonstrated to be pathogenic, G261R [21,28,29] and W257R [30,31]. To find these regions, we graphed the frequency of VUS, likely pathogenic, and pathogenic variants derived from ClinVar (NCBI) against the primary sequence of GCK on the X-axis (Figure 1). We chose low-pathogenic regions at positions 119-132 and 412-427 as the experimental regions. We chose a high pathogenic region at positions 257-262 as the control region.

**Figure 2:**
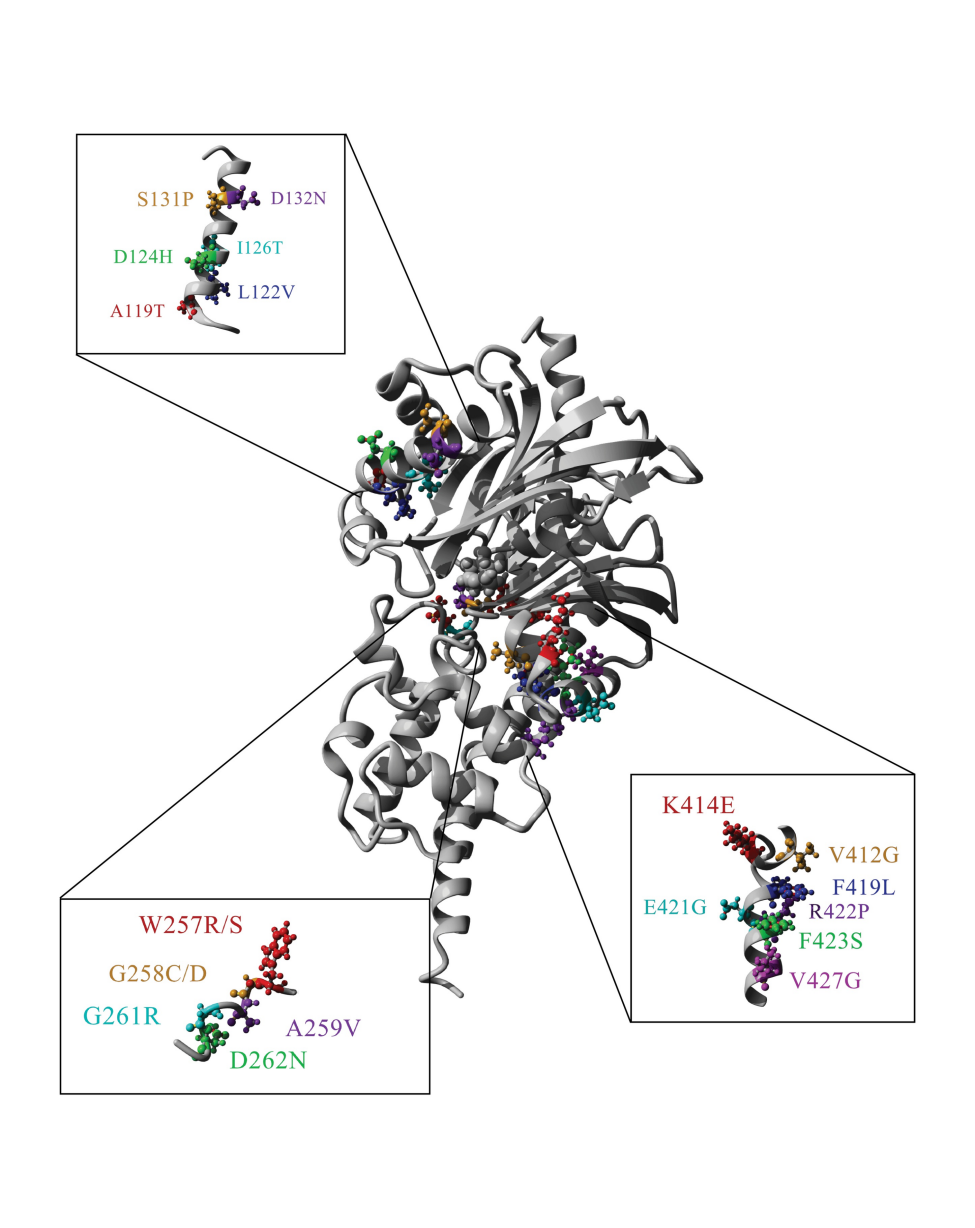
Three-dimensional representation of variants analyzed. The variants identified in Figure 1 are in three regions of glucokinase. Variants in region 119-132 are located in the a3 helix (upper left). Variants in region 257-262 are located between b8 and b6 sheets and the subsequent turn (bottom left). Variants in region 412-427 are in the a12 helix and b9 sheet (bottom right). The secondary structure was described previously [2].

The experimental regions are located in solvent-exposed portions of the protein, whereas the control region is located deep in the protein near the active site (Figure 2). One known pathogenic variant, W257R, is adjacent to an active site residue, E256 [4].

In vitro analysis

**Figure 3:**
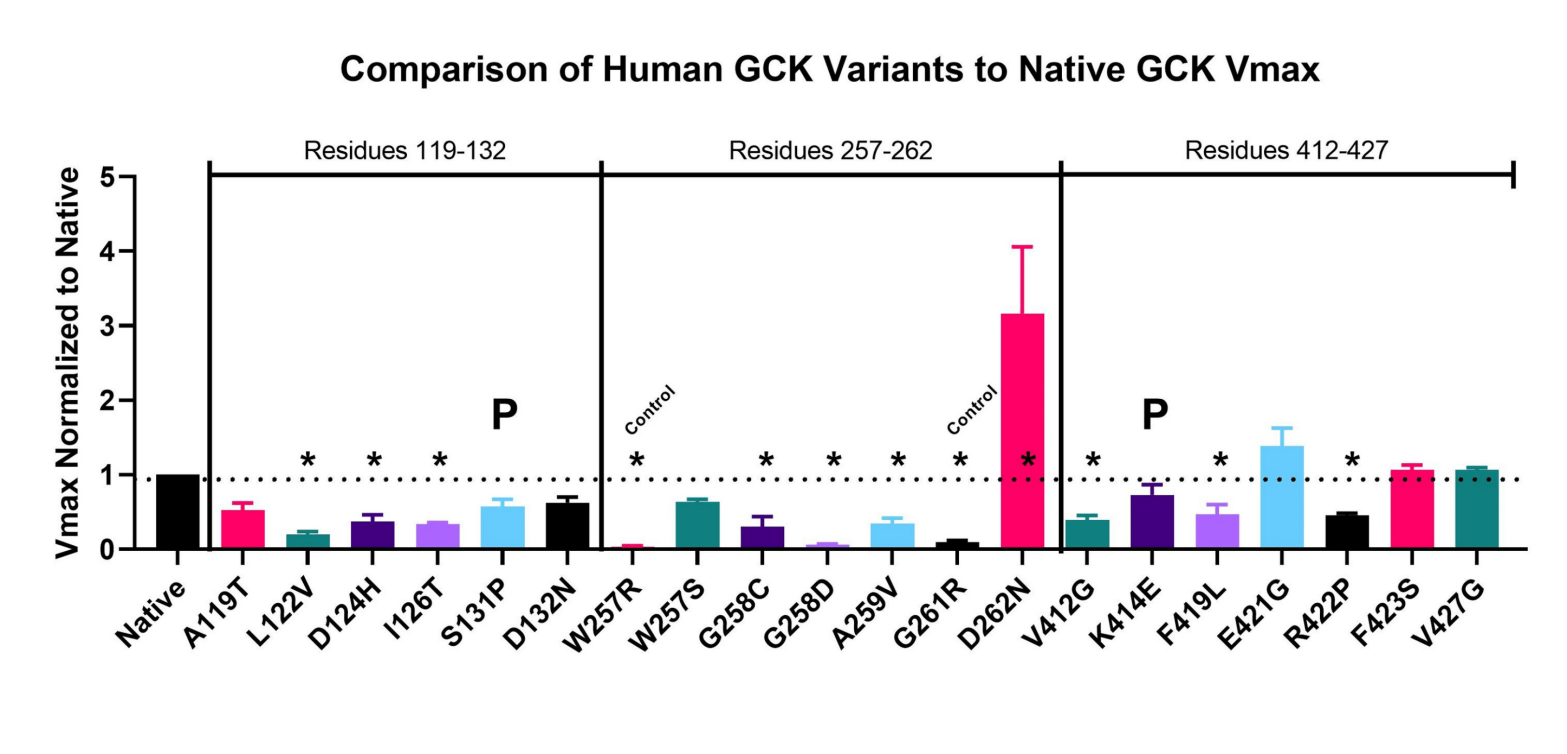
In vitro comparison of human glucokinase (GCK) variants. The Vmax values were obtained for each recombinant human glucokinase (GCK) variant protein. Values are normalized to native protein Vmax. Asterisks indicate p < 0.01. The W257R and G261R variants are negative controls known to have little activity based on previous publications [21,28-31].

To analyze these variants, we chose to compare the in vitro activity of recombinant protein to a variety of predictive computer analyses. Figure 3 demonstrates the activities of each variant tested in three separate experiments each. Within each experiment, the variant Vmax was compared to the native protein Vmax to normalize the activity. The native protein served as a positive control, whereas known pathogenic variants, W257R and G261R, were used as negative controls. The almost complete lack of activity of the negative controls shows that the purification techniques were efficient enough to eliminate all bacterial GCK activity that may create background activity in the assay. The variants that had activity that was not significantly different compared to native protein were A119T, S131P, D132N, W257S, K414E, E421G, F423S, and V427G.

We initially predicted that almost all of the VUS in the low-pathogenic variant regions would have a lower frequency of VUS with low Vmax activities. Two out of five VUS in the 119-132 region had non-significant Vmax values compared to native protein. Three out of six VUS in the 421-427 region had non-significant Vmax values compared to native protein. Interestingly, both variants that had been designated as pathogenic or likely pathogenic on ClinVar, S131P and K414E, were not significantly different from native protein. This suggests that other factors outside of enzymatic rate contribute to clinical pathogenicity.

In contrast to the experimental regions, in the 257-262 pathogenic region near the active site, only one out of five VUS exhibited non-significant Vmax values compared to the native protein. Interestingly, one of the VUS showed a significant increase in activity. It is unknown whether the increase in activity indicates a significant change in structure that ultimately results in a pathogenic phenotype through a mechanism outside the enzymatic rate.

In silico analysis

**Figure 4:**
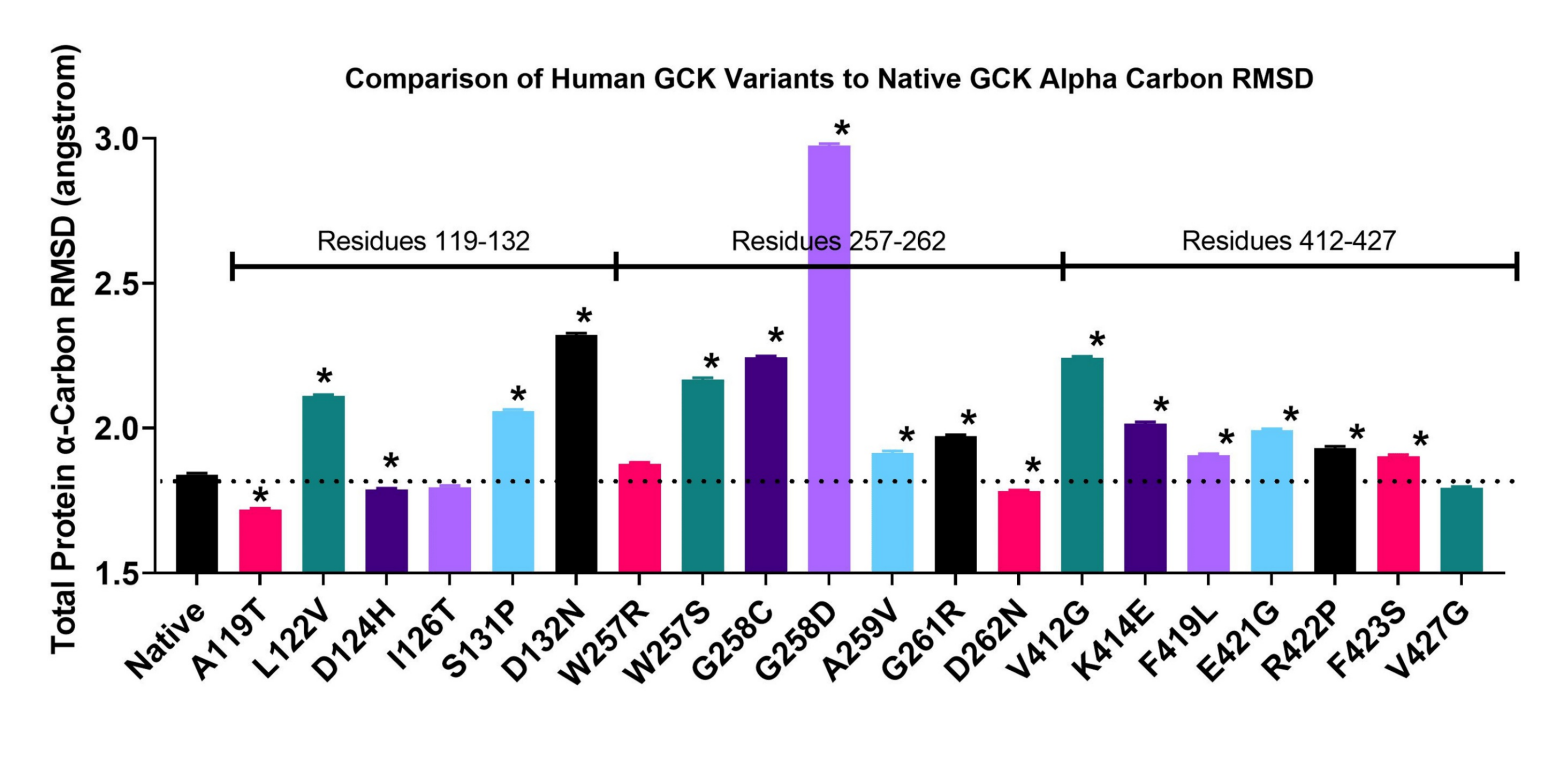
In silico glucokinase (GCK) variant comparison of alpha carbon root-mean-squared deviation (RMSD). Molecular dynamics simulations were performed with the glucokinase variants for 20 ns. The root-mean-squared deviation (RMSD) values for three trials over the 10-20 ns time range were averaged. The averages were then used for statistical comparison to the native protein. Asterisks indicate p < 0.01.

The VUS were then compared using molecular dynamics simulation (Figure 4) [32,33]. A three-dimensional GCK protein structure was generated from the complete primary protein sequence through homology modeling using the YASARA program [10,23,24]. The three-dimensional structures for each variant were created by replacing the respective residue in the native protein structure. All structures were then run through three simulation trials for 20 ns each. The total protein RMSD was averaged for the last 10 ns of the simulation and compared statistically. The first 10 ns of the simulation were excluded from the calculations to allow the protein to acclimate to the simulation conditions. Only three variants were not statistically different compared to the native protein: I126T, W257R, and V427G. Comparing the RMSD data of these variants to the in vitro results, we see that I126T and W257R had decreased enzymatic activity, whereas V427G had activity equivalent to native protein. W257R has also been determined to be clinically pathogenic, as indicated on ClinVar.

Most RMSD results showed a higher trend than the native protein RMSD. No observed correlation exists between enzymatic activity and RMSD value. The structure of each variant GCK protein at the end of the simulation was compared to the native post-simulation structure. No consistent change in protein structure correlated with the enzymatic activity results (data not shown).

**Figure 5:**
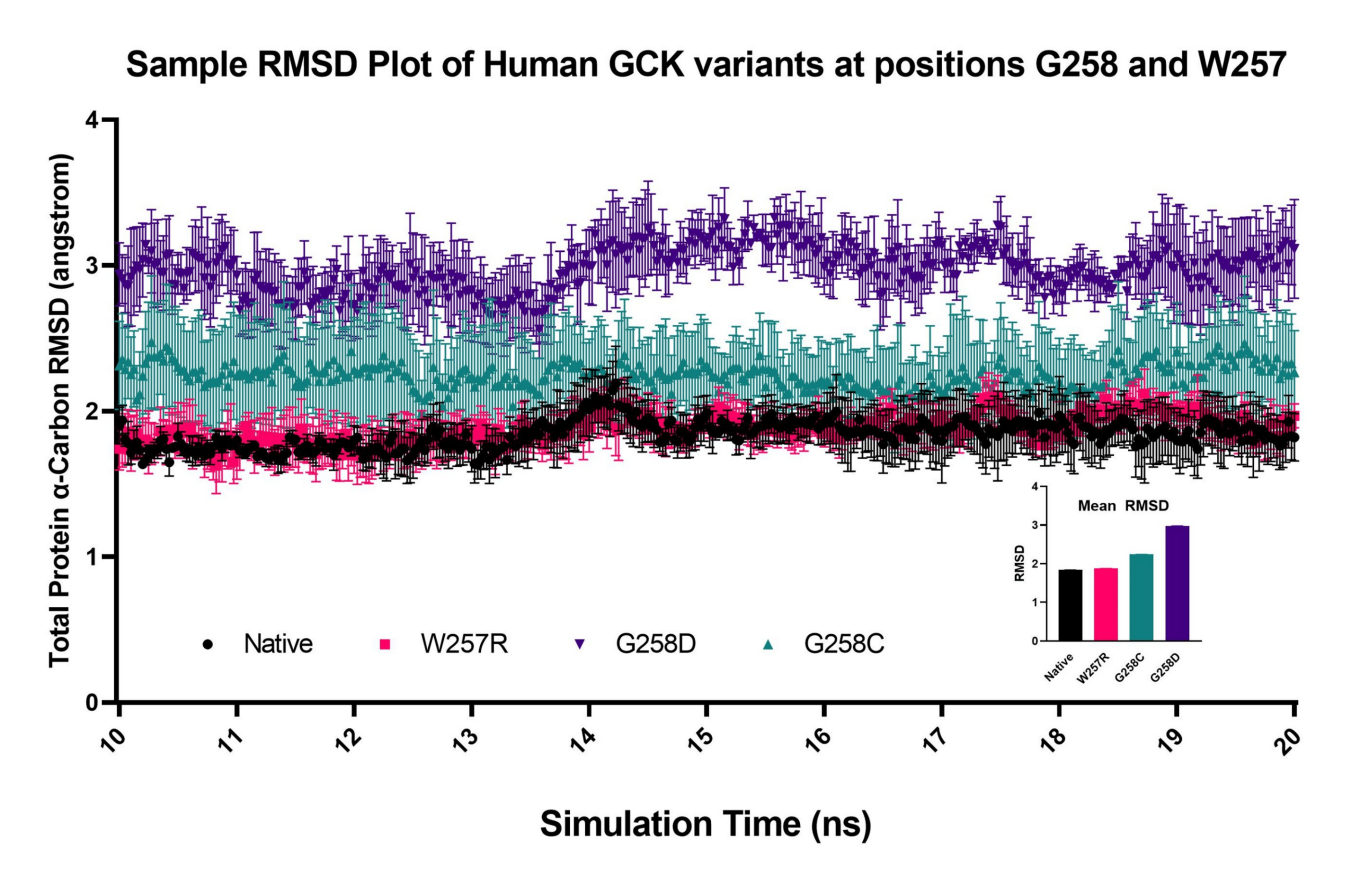
Comparison of total glucokinase (GCK) alpha carbon root-mean-squared deviation (RMSD) during simulation. This figure demonstrates sample root-mean-squared deviation (RMSD) plots for the molecular dynamics simulation over the 10-20 ns time range. There is a representative for high RMSD change (G258D), intermediate change (G258C), and no change (W257R) relative to native protein. The error bars represent the standard error of the RMSD values for three trials. The inset shows the average RMSD values for the variants, as demonstrated in Figure 4.

Figure 5 shows an example of the RMSD data over the 10-20 ns time frame for a variant that showed no difference, W257R, a variant with a moderate difference, G258C, and a variant with a large difference, G258D, compared to the native protein. The data shown in this figure show that the differences between the variant RMSD were due to a consistent change in structure over the 10-20 ns time frame and not due to dramatic RMSD fluctuations over shorter periods of time.

**Figure 6:**
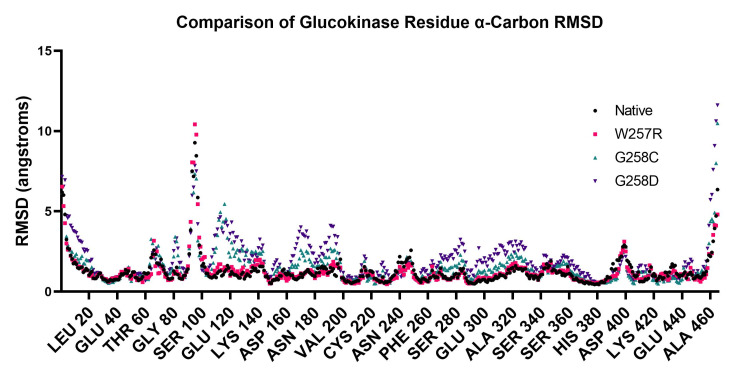
Comparison of alpha carbon root-mean-squared deviation (RMSD) of glucokinase (GCK) residues during simulation. The molecular dynamics simulation root-mean-squared deviation values for each residue's alpha carbon were compared using the same variants used in Figure 5.

Figure 6 demonstrates the RMSD for each residue in each variant. The RMSD differences occur in specific regions (e.g., around residues 112 and 170), whereas other regions do not have significant changes (e.g., around residues 40 and 380). These changes are roughly proportional to the total protein RMSD changes.

**Figure 7:**
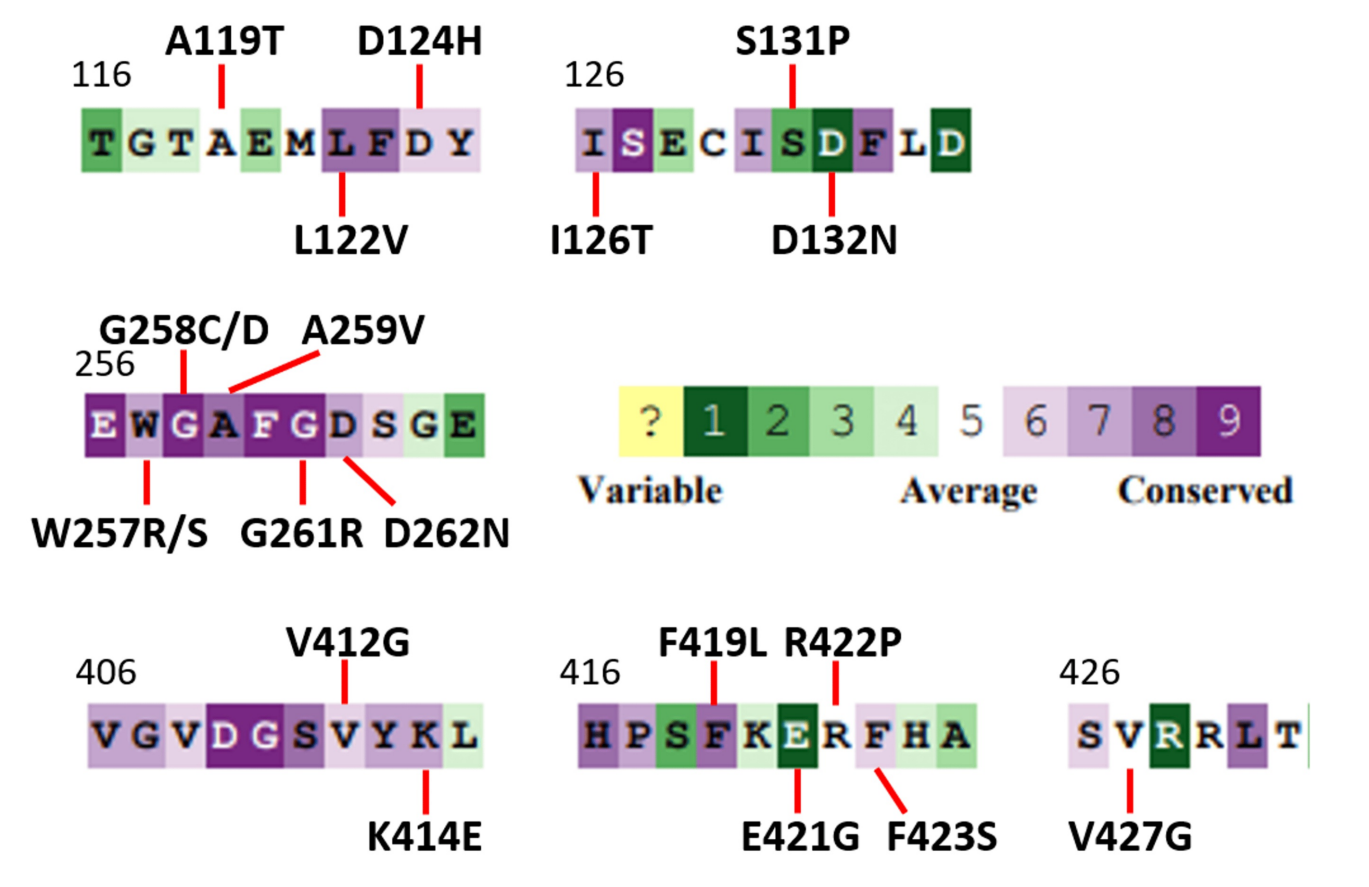
Conservation of the tested variants. This is a visual representation of the conservation of the variant residues as calculated by ConSurf [14]. Highly conserved residues are indicated by purple, and variable residues are indicated by green. Numbers indicate the primary sequence position.

ConSurf was used to analyze the conservation at the residue positions of the tested variants [14]. Figure 7 shows the variants and the color-coded conservation result at each position. The two experimental regions as a whole had more variable residues compared to the control region, which was largely conserved, likely due to its proximity to the active site.

According to Figure 3, six VUS had non-significant changes in Vmax and thus can be considered putative benign variants. Nine VUS are significantly different compared to native protein and are considered putative pathogenic variants. With the VUS tested, four variants were identified previously as pathogenic. One VUS, D262N, was tested with higher enzymatic activity. This higher activity likely rules it out as pathogenic for MODY, but it may contribute to hyperinsulinemic hypoglycemia. This difference in activity makes it unique among the variants analyzed and, therefore, was not used in the comparison for MODY pathogenicity. In Table 1, the in vitro data are used as a reference for pathogenicity. Here, the variant Vmax values that are significantly different compared to native protein are marked in column A as "SD."

**Table 1:** Comparison of in vitro results to bioinformatic results. The table compares the in vitro Vmax averages from Figure 3 to results from predictive programs. The second column indicates which variants are VUS or pathogenic, as designated in ClinVar. The "control" variants are pathogenic variants proven to have low GCK activity in vitro. The Vmax values are indicated in the next column, followed by column A, indicating whether the Vmax results are statistically different, SD, or not significantly different, NS, using two-way ANOVA, p<0.01. The "?" for the D262N variant indicates the questionable interpretation of pathogenicity. The ConSurf row shows the conservation score (negative values=more conserved; positive values=more variable). Column B indicates if the residue position is conserved "C" because the value is less than -0.4, not conserved "NC" because the value is more than -0.4, or the variant change is not tolerated "NT." The iStable column values are followed by column C, which indicates if the variant is predicted to be pathogenic "P" (< -0.8) or benign "B" (> -0.8). The EVE values are followed by column D, which indicates if the variant is predicted by the evolutionary model of variant effect (EVE) to be pathogenic "P," benign "B," or was unable to make a determination of pathogenicity "ND." The PredictSNP confidence values were followed by column E, which indicates if PredictSNP indicates if the variant is pathogenic "P," benign "B." The change in binding energy values between native and variant proteins is indicated, followed by column F, which indicates if the variant is predicted to be pathogenic "P" (>0.2 kcal/mol) or benign "B" (<0.2 kcal/mol). In columns B-F, the "-" indicates the pathogenicity determined by the in vitro data or published results does not match the predictive result; "+" indicates the variant results do match; "X" indicates the determination of pathogenicity of the D262N variant cannot be determined due to a high Vmax. The last row indicates the number of matches for each predictive program.

Variant	Designation	Relative Vmax	A	ConSurf	B	iStable	C	EVE	D	Predict SNP	E	Binding Energy D	F
A119T	VUS	0.53	NS	-0.1	+/NC	-1.11	-/P	0.20	+/B	75%	+/B	0.01	+/B
L122V	VUS	0.20	SD	-1.2	+/C	-1.57	+/P	0.39	ND	76%	+/P	0.02	+/P
D124H	VUS	0.37	SD	-0.5	+/C	-0.95	+/P	0.53	ND	72%	+/P	0.00	-/B
I126T	VUS	0.34	SD	-0.7	+/C	-1.94	+/P	0.66	+/P	87%	+/P	0.03	+/P
S131P	Pathogenic	0.58	NS	1.2	+/NT	-0.83	+/P	0.39	ND	60%	-/B	0.12	+/P
D132N	VUS	0.63	NS	1.2	+/NC	-0.86	-/P	0.12	+/B	83%	+/B	0.00	+/B
W257R	Control	0.04	SD	-0.8	+/C	-2.26	+/P	0.83	+/P	87%	+/P	0.11	+/P
W257S	VUS	0.63	NS	-0.8	-/C	-2.60	-/P	0.45	ND	76%	-/P	-0.04	+/B
G258C	VUS	0.30	SD	-1.3	+/C	-0.88	+/P	0.63	ND	87%	+/P	0.29	+/P
G258D	VUS	0.06	SD	-1.3	+/C	-1.68	+/P	0.62	ND	87%	+/P	0.07	+/P
A259V	VUS	0.35	SD	-0.9	+/C	-0.96	+/P	0.56	ND	87%	+/P	0.00	-/B
G261R	Control	0.09	SD	-1.2	+/C	-1.37	+/P	0.60	ND	87%	+/P	-0.02	-/B
D262N	VUS	3.17	?	-0.6	X/C	-0.02	X/B	0.29	X/B	60%	X/B	0.04	X/P
V412G	VUS	0.39	SD	-0.4	+/C	-1.75	+/P	0.62	ND	87%	+/P	0.00	-/B
K414E	Pathogenic	0.73	NS	-0.6	+/C	0.26	-/B	0.20	-/B	63%	-/B	-0.01	-/B
F419L	VUS	0.47	SD	-0.9	+/C	-1.49	+/P	0.28	-/B	87%	+/P	0.02	+/P
E421G	VUS	1.39	NS	1.3	+/NC	-1.92	-/P	0.12	+/B	83%	+/B	0.00	+/B
R422P	VUS	0.46	SD	0.1	+/NT	-1.05	+/P	0.44	ND	87%	+/P	0.00	-/B
F423S	VUS	1.07	NS	-0.3	+/NC	-2.54	-/P	0.17	+/B	61%	-/P	0.06	-/P
V427G	VUS	1.06	NS	-0.1	-/NT	-2.86	-/P	0.64	ND	76%	-/P	0.00	+/B
Total corresponding predictions			-	-	17	-	12	-	6	-	14	-	12

Table 1 compares the in vitro results with other computational biology resources, where ConSurf appears to correlate well with the in vitro results. In column B, the variants at conserved positions, C, and variant changes that were not tolerated, NT, were considered pathogenic, whereas the variants at positions that were not conserved, NC, were considered benign. The + and - indicate if the ConSurf results match the in vitro data. Of the 16 VUS tested, 13 VUS matched the ConSurf results, and D262N was difficult to interpret. Nine VUS had significantly different Vmax results and, simultaneously, had a ConSurf score below -0.4 or a positive ConSurf score, but the variant residue was not tolerated, R422P. The remaining four VUS had non-significant Vmax differences and a ConSurf score above -0.4, A119T, D132N, E421G, and F423S. As noted above, the pathogenic variants S131P and K414E did not have significantly different Vmax values. In the case of S131P, ConSurf determined that proline at that position would not be tolerated. In the case of K414E, ConSurf had a value of -0.6 and was thus under the -0.4 threshold. The main exceptions to this correlation were W257S, which had non-significant Vmax values and yet had a -0.8 ConSurf value, and V427G, which had a non-significant Vmax value and yet ConSurf predicted that a glycine would not be tolerated at that position.

iStable had a moderate correlation of stability and pathogenicity. Using a scoring system that maximized the matches of VUS pathogenicity to the iStable scores, the threshold for pathogenicity was set to -0.8. With this system, most variants were scored as pathogenic, with only D262N and K414E scored as benign. With this system, 12 variants had scores that matched the in vitro data.

The EVE program was not able to determine pathogenicity for 11 of the 20 variants tested. However, it was able to designate four of the six putative benign VUS as benign, but only one putative pathogenic VUS was designated as pathogenic. Only one known pathogenic variant, W257R, was designated as pathogenic, and the hyperactive variant D262N was designated as benign.

PredictSNP correlated well with the in vitro results. Three of the six putative benign VUS were identified as benign by the program, whereas all nine of the putative pathogenic VUS were designated as pathogenic. Two of the known pathogenic variants were designated as pathogenic, and the D262N VUS was designated as benign.

Using the final coordinates of the protein after the 20-ns molecular dynamics simulation, we used YASARA to predict the binding energy of glucose to the active site of each variant protein. The last column in Table 1 shows the difference in ligand binding energy between the variant and native proteins. A 0.02 kcal/mol cutoff was used to maximize the number of matches with the in vitro data. Five of the six putative benign VUS had little change in binding energy. Five of the nine putative pathogenic VUS exhibited an increase in binding energy. Two of the known pathogenic variants had an increase in binding energy.

## Discussion

The original hypothesis was that regions with low-pathogenic variant rates would have more benign VUS. The combined rate of benign VUS, as indicated by non-significant differences compared to the native protein, was five benign out of 11 VUS (45%) compared to the one benign VUS of five VUS (20%) tested in the high pathogenic variant range. Although the number of VUS tested was low, this suggests that the VUS from regions with fewer pathogenic variants will be more likely benign compared to more pathogenic regions.

This report is one of the few analyses of GCK that combines in vitro detection of variant protein activity with in silico variant pathogenicity prediction methods. Most of the VUS tested were not described in previous publications. The I130T VUS was characterized using in vitro methods [34]. The D133N VUS was characterized using predictive programs such as PolyPhen2, SIFT, MutationTaster, and CADD [35]. Recently, Gersing et al. reported that a mutational screen of human GCK was performed using a yeast complementation assay [36]. Most of the VUS were determined to be pathogenic with this yeast method. Of the 16 VUS that we tested, Gersing et al. reported that 12 VUS were pathogenic, two were benign (D132N and E421G), and two were not scored (D124H and G258C) [36]. Of the nine VUS that we found to have statistically different activities, seven were also scored as pathogenic by Gersing et al. (L122V, I126T, G258D, A259V, V412G, F419L, and R422P). Gersing et al. did not use predictive programs or molecular dynamics simulation, but they did perform a conservation analysis to compare to their yeast data [36].

Although ConSurf successfully predicted the pathogenicity of the GCK variants tested, it was not 100% correct. At the same time, the other methods available, including the methods used here, are not inferior. They may just identify different ways a variant can be pathogenic. Ideally, there should be a method that correctly identifies pathogenicity every time to allow for analysis of a patient genotype that has novel or rare variants. We propose that there is likely a combination of programs that will accurately predict pathogenicity. This might involve an algorithm that takes into account the type of residue changed, the location of the residue within the protein, the secondary structure surrounding the residue, etc. There are many different programs that can be compared using the method described here [13]. Unfortunately, we did not have a GCK mutational scanning method at the time that would allow us to analyze the programs' predictive power over the entire protein.

We are reporting the enzymatic activities of GCK variants. A limitation of our study is that our data do not address any variant effects that may not directly affect enzymatic activity. For example, a nucleotide variation can disrupt a splice junction [37]; the protein variation can interfere with ligand/protein partner binding; the variation may lead to mislocalization of the protein within the cell; the variant can destabilize the protein, leading to aggregation or degradation, etc. [38]. For instance, the variants' response to regulation by GCK regulatory protein could have also been tested [39]. It was beyond the scope of this research to investigate every way that the GCK function may be affected by a variation.

A strength of the study was the combination of in vitro data with multiple in silico analyses. Most reports focus on one or the other type of variant analysis. This report used molecular dynamics simulation, ConSurf, iStable, EVE, PredictSNP, and predicted binding energy. All of these methods have their strengths and weaknesses. Eventually, a consensus may be established on which predictive method works best for most variants.

The D262N variant was an outlier because it had Vmax activity that was higher than that of the native protein. This higher activity would catalyze the phosphorylation of glucose at a higher rate, which may ultimately result in insulin secretion at lower glucose concentrations [40]. Genetic variation databases have no data regarding the physiological impact of the D262N variation and thus categorize it as a VUS [41,42]. Other activating GCK variants have been discovered that cause persistent hyperinsulinemic hypoglycemia, which is characterized by low blood glucose and high blood triglyceride levels. Previously identified activating variations include A456V, V455M, T65I, and W99R [40,43,44]. GCK mutational screen data indicate that the D262N variant has a slight decrease in activity (0.58 score out of 1.0) [36]. Further investigation would be needed to confirm D262N as an activating GCK variation.

Part of the purpose of this report is to try to identify pathogenic GCK variants from the pool of VUS. The problem encountered is that there is no easy way to correlate the in vitro results with the phenotype, especially when the in vitro results indicate a gain-of-function or only a mild loss-of-function. When analyzing outlier variants such as the D262N variant, should the variants be considered pathogenic because their activity is statistically different than that of the native protein? Along the same line, at what point should a difference in activity of either inactivating or activating variants be considered pathogenic? In this study, we used statistical significance as the criterion for pathogenicity. Instead, should a threshold of enzymatic activity be determined using clinical data? Of course, a variant with a mild reduction of protein activity may have some physiologic effect, but it likely would not have the same phenotype as a variant with a 95% reduction in activity. These are all questions that will need to be answered regarding future variant pathogenicity prediction [32].

## Conclusions

This report compares the enzymatic rates of recombinant GCK protein to various computational biology techniques to assess pathogenicity. The VUS within low pathogenicity regions of the protein were compared to the VUS within high pathogenicity regions. Of the VUS within low pathogenicity regions, L122V, D124H, I126T, V412G, F419L, and R422P had significantly decreased activity. A119T, D123N, E421G, F423S, and V427G did not have a significant decrease in activity. G258C, G258D, and A259V had significantly decreased activity, while W257S did not have a significant decrease in activity. S131P, W257R, G261R, and K414E were used as pathogenic controls. If the recombinant protein activities serve as a definitive measurement of one aspect of pathogenicity, then we can compare the other bioinformatic resources to determine their efficacy at predicting changes in enzymatic activity. One variant, D262N, had a statistically significant increase in enzymatic activity. Because we could not determine if this should be classified as pathogenic or benign, we excluded this variant from the analysis. Of the variants tested, MDS RMSD differences identified 12 of the 19 variants correctly, ConSurf identified 17 of the 19 variants correctly, iStable identified 12 of the 19 variants correctly, EVE identified six of the 19 variants correctly, PredictSNP identified 14 of the 19 variants correctly, and calculating the change in binding energy identified 12 of the 19 variants correctly. These comparisons suggest that ConSurf was the best at predicting the change of activity of the set of variants that we tested.
